# Identifying and Assessing Interesting Subgroups in a Heterogeneous Population

**DOI:** 10.1155/2015/462549

**Published:** 2015-08-03

**Authors:** Woojoo Lee, Andrey Alexeyenko, Maria Pernemalm, Justine Guegan, Philippe Dessen, Vladimir Lazar, Janne Lehtiö, Yudi Pawitan

**Affiliations:** ^1^Department of Medical Epidemiology and Biostatistics, Karolinska Institutet, 17177 Stockholm, Sweden; ^2^Department of Statistics, Inha University, Incheon 402-751, Republic of Korea; ^3^Department of Microbiology, Tumour and Cell Biology, Bioinformatics Infrastructure for Life Sciences, Science for Life Laboratory, Karolinska Institutet, 17177 Stockholm, Sweden; ^4^Department of Oncology and Pathology, Science for Life Laboratory, Karolinska Institutet, 17121 Solna, Sweden; ^5^Genomics, Institut Gustave Roussy, F-94805 Villejuif, France

## Abstract

Biological heterogeneity is common in many diseases and it is often the reason for therapeutic failures. Thus, there is great interest in classifying a disease into subtypes that have clinical significance in terms of prognosis or therapy response. One of the most popular methods to uncover unrecognized subtypes is cluster analysis. However, classical clustering methods such as *k*-means clustering or hierarchical clustering are not guaranteed to produce clinically interesting subtypes. This could be because the main statistical variability—the basis of cluster generation—is dominated by genes not associated with the clinical phenotype of interest. Furthermore, a strong prognostic factor might be relevant for a certain subgroup but not for the whole population; thus an analysis of the whole sample may not reveal this prognostic factor. To address these problems we investigate methods to identify and assess clinically interesting subgroups in a heterogeneous population. The identification step uses a clustering algorithm and to assess significance we use a false discovery rate- (FDR-) based measure. Under the heterogeneity condition the standard FDR estimate is shown to overestimate the true FDR value, but this is remedied by an improved FDR estimation procedure. As illustrations, two real data examples from gene expression studies of lung cancer are provided.

## 1. Introduction

Biological heterogeneity is common in many diseases; heterogeneity complicates clinical management, as it is often the reason for prognostic and therapeutic failures. Thus, there have been many attempts to classify a disease into subtypes with anticipation that different subgroups are associated with different clinical significance in terms of prognosis or therapy response (e.g., [1, 2]). A significant progress in designing efficient specific treatments can be achieved if novel clinically relevant subtypes are found.

One of the most popular methods for finding unrecognized subtypes is cluster analysis. However, classical clustering methods such as *k*-means clustering or hierarchical clustering are not guaranteed to produce clinically interesting subtypes because the main statistical variability could be dominated by genes not associated with interesting clinical phenotypes. Furthermore, it could be that prognostic factors shared within a subgroup do not have any important role in other subgroups. Thus, the association between prognostic factors and a clinical phenotype is attenuated and not detectable in the whole population. To address these problems, we extend the standard clustering algorithm to find interesting subgroups in the sense that within the subgroup we can find factors (in this paper: genes) strongly associated with the clinical phenotype. This idea can perhaps be illustrated more clearly as follows: suppose that *Y* is an outcome (e.g., relapse) and *X* is a randomized treatment; it is common to search for a subgroup for which the treatment effect is largest. In effect we are searching for factors *Z* that have significant interactions with *X*, such that a subgroup defined by *Z* will have a large treatment effect on *Y*. A unique point in our current application is that both *X* and *Z* are given by the same set of gene expression data. Also, we allow complex subgroups to be discovered by a clustering method, which makes the process distinct from the standard interaction analysis.

Given a set of gene expression matrix, our goal of cluster analysis is to group patients and genes into subgroups that convey biological or clinical significance. This task can be translated to the biclustering problem. Biclustering methods attempt to simultaneously cluster both patients and genes with the goal of finding subsets of rows and columns in the expression matrix. Cheng and Church [3] firstly introduced biclustering to gene expression analysis. For reviewing the details of biclustering algorithms, see [4]. As Nowak and Tibshirani [5] noticed, however, most of biclustering algorithms tend to be dominated by groups of highly differentially expressed (DE) genes that may not be relevant to the biological process in question. In other words, irrelevant genes with strong signal can mask genes of highest biological relevance. Furthermore, iterative optimization methods adopted in biclustering algorithms depend on initial conditions. To overcome these limitations, we develop an extensive clustering search algorithm to find molecular subtypes (CAMS) based on clustering of patients with partially similar mRNA profile. CAMS is able to uncover the structures arising from relevant genes that may not be highly expressed but moderately expressed within each subtype.

CAMS produces many subtypes. For each subtype, *t*-statistics comparing two distinct phenotype groups (e.g., relapsed/not relapsed) are computed for whole genes and false discovery rate (FDR) estimate is used to correct for multiple comparisons. The number of genes having small FDR estimates (say, less than 0.1) is the basis for assessing the importance of the subtypes. In real data analysis, however, it is a common occurrence in heterogeneous populations that *P* value distributions of the two-sample *t*-statistics show substantial shortage of small values compared to the uniform distribution [6]. If we ignore this effect we would miss potential discoveries by overestimating FDR. Since subtypes produced by CAMS still can be heterogeneous, it is crucial to study how the molecular heterogeneity of distinct subtypes affects the FDR estimate. In this paper, we introduce unobserved group (or latent group) variables into a simple model for gene expression and see how the heterogeneity induced by the unobserved group leads to the depletion of small *P* values even when there are many significant signatures. Thus, without considering this underlying heterogeneity, the use of standard FDR estimate might hide promising discoveries. To resolve this problem, we develop an improved FDR estimation procedure to address the heterogeneity in a dataset.

In estimating FDR, the use of correct null density function is critical. Efron [7] considered three issues that substantially affect the null density estimate in computing FDR: (1) a large proportion of genuine but uninterestingly small effects, (2) hidden correlations, and (3) unobserved covariates. Many researchers have studied how they affect the standard FDR estimate [7–9]. In particular, possible connections between unobserved covariates and FDR have been explored in [6, 10]. Leek and Storey [6] showed numerically that the small *P* values range from being inflated to depleted depending on the configuration of the unobserved covariates. They developed the so-called surrogate variable analysis (SVA) for capturing heterogeneity induced by the unobserved covariates and studied how SVA affects FDR estimate. Stegle et al. [10] considered a Bayesian method to account for hidden confounding variation in expression quantitative trait loci (QTLs) and showed that the method found additional expression QTLs in real datasets. However, their approaches were suggested to study the attenuated relationship by heterogeneity between a measured variable of interest and clinical outcomes, while we focus on finding submerged subtypes by heterogeneity. The novel contributions of this paper are (1) to explain how the heterogeneity induced by unobserved group leads to the depletion of small *P* values analytically, (2) to analyze the bias of standard FDR estimates under the heterogeneity, and (3) to develop an improved FDR estimation procedure. With these in mind a FDR-based measure is considered to assess findings from a novel clustering procedure. This is illustrated using two datasets on lung cancer patients.

The rest of this paper is organized as follows. In Section 2, we describe the implementation details of CAMS. A brief review of notations and a standard FDR estimation method are given in Section 3, and it is analytically shown that the hidden subgroup in the population can induce a bias of standard FDR estimate in Section 4. We propose a FDR estimation procedure resolving the bias problem and show how to assess clustering results from CAMS with it in Sections 5 and 6.  Section 7 includes two real data applications and is followed by concluding remarks.

## 2. Clustering Algorithm for Finding Molecular Subtypes

Consider a set of gene expression profiles from a group of cancer patients. The premise behind CAMS is that the novel molecular information on cancer heterogeneity is hidden in the gene expression profiles. To uncover the heterogeneity, CAMS implements a two-dimensional clustering “patients versus genes" extensively. The full algorithm is given in Algorithm 1.

**Figure 1:**
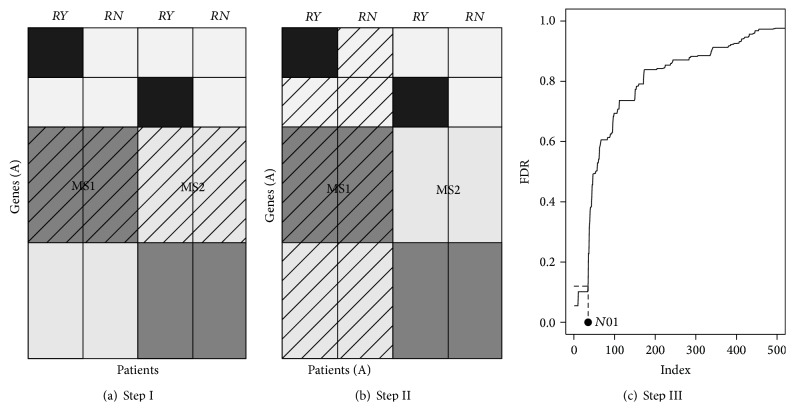
(a) Step I is clustering of genes. Genes (A) (shaded region) are grouped and will be used as a subtype identifier in the downstream analysis. (b) Step II is clustering of patients using Genes (A) (i.e., a gene-set obtained from Step I). Here, MS1 (a set of patients, shaded regions) is obtained as a subtype. (c) Step III shows how *N*01 is obtained from the FDR curve. We count the number of genes having FDR < 0.1. We repeat implementing (a), (b), and (c) across different clustering results extensively. Thus, no shaded columns in (b) will be covered subsequently.

We first explain the clustering steps of CAMS graphically in Figures 1(a) and 1(b). In the two figures, a set of gene expression profiles as a matrix with rows corresponding to genes and columns corresponding to patients is graphically represented. For illustrative purposes, we designed the following simple model.

- It has two observed groups: for example, relapse yes (*RY*) and relapse no (*RN*) groups.
- It has two unobserved groups: the first two columns correspond to molecular subtype 1 (MS1) and the remaining two columns correspond to molecular subtype 2 (MS2). This information is unknown to the researchers.
- Some genes (marked in black) affect relapse within a MS.

The two key clustering steps of CAMS are as follows. Step I is clustering of genes. This step identifies several sets of genes having similar profiles across the patients. For example, in Figure 1(a), gene-set A (shaded region) is grouped and this will be used as a subtype identifier in next step. Step II is clustering of patients using gene-set A only. This step produces a subgroup of patients (individuals belonging to the shaded region of Figure 1(b)) with a common expression profile for gene-set A. Note that this subgroup is homogeneous in terms of the identified set of genes from the first step but can show distinct expression profiles between* RY* and* RN* on the other set of genes (e.g., genes marked in black). Thus, we hope that, within the subgroup of patients, good prognostic models can be constructed.

Technical description for CAMS is given as follows. In Step I, the set of gene probes on the microarray chip is grouped via hierarchical clustering. This is implemented using* hclust* in *R*. All the hierarchical clustering in this paper uses complete linkage method and Euclidean metric. This hierarchical clustering procedure is applied to disjoint subsets (*S*) of *m* all available gene probes due to computational limit (e.g., *m* = 41000 gene probes in the lung cancer dataset). For example, if *S* = 10, our procedure makes 10 disjoint subsets of gene probes and each subset has *m*/10 gene probes sampled from the whole list. Then the whole list is systematically covered by applying the clustering to *S* subsets sequentially. To allow various groupings of gene probes under different environments, we shuffle the whole list of gene probes several times. The number of clusters (*C*) from each subset *S* varies on a vector of fixed numbers. For example, if *C* = (2,3, 4,5, 6,7, 8,9, 10), then 9 different cluster analysis results are considered in the downstream analysis. Thus each gene probe could participate in different clustering solutions, from very large (>500 probes) to small sets. These clustering results can be used as subtype identifiers in next step.

In Step II, the same hierarchical clustering method is applied to cluster the patients by using each subtype identifier separately. Then the dendrogram is cut at the highest level where the clusters contain more patients than the threshold. Each subset of patients is treated as a candidate subtype.

When the clustering steps of CAMS are performed, only some of found cancer subtypes would be true discoveries. To assess whether subtypes are promising or not, *t*-statistics comparing two distinct phenotype groups (e.g., relapsed/not relapsed) within each subtype are computed for whole genes and the number of genes having small FDR estimates (say, less than 0.1) is calculated based on the *P* values of the *t*-statistics. However, the effect of the molecular heterogeneity on this assessment has not been explored in detail. To deal with this issue, we first review a standard FDR estimation method below.

## 3. Notation and Standard FDR Estimation

In this section some basic notations are introduced to give a formal definition of FDR. For clarity and simplicity, we will limit our discussion to the most common problem of finding differentially expressed (DE) genes between two biological conditions. Let *z* be a certain statistic to compare the mean log-expression level. The distribution of observed statistics *z* follows a mixture model

(1) $$\begin{array}{c} f\left(z\right)=\pi_{0}f_{0}\left(z\right)+\left(1-\pi_{0}\right)f_{1}\left(z\right), \end{array}$$

where *π*_0_ is the proportion of truly nondifferentially expressed (non-DE) genes and *f*_0_(*z*) and *f*_1_(*z*) are the density functions of *z* for non-DE and DE genes, respectively.

Suppose we test *m* genes with corresponding statistics *z*_1_,…, *z*_*m*_. Let *P*_1_,…, *P*_*m*_ be the ordered *P* values from *m* test statistics. For a fixed critical value *c*, we define the number of non-DE genes declared DE and the number of genes declared DE as

(2) V(c)=∑iI(Pi≤c,i∈Null),R(c)=∑iI(Pi≤c),

where *I*(·) is the indicator function. Then, the false discovery proportion (FDP) is defined as

(3) $$\begin{array}{c} \text{FDP}\left(c\right)=\frac{V\left(c\right)}{R\left(c\right)}, \end{array}$$

except in the case of *R*(*c*) = 0, in which case we just set FDP(*c*) = 0. The FDP is random proportion of false discoveries among the genes declared to be DE. The standard FDR is the marginal average of the FDP; namely, FDR(*c*) = *E*(FDP(*c*)).

The standard estimate of FDR [8, 11] as a function of the ordered *P* values is given by

(4) $$\begin{array}{c} \hat{\text{FDR}}\left(P_{k}\right)=\frac{m{\hat{\pi }}_{0}P_{k}}{k}. \end{array}$$

Monotonicity is imposed by taking the cumulative minimum over $\hat{\text{FDR}}\left(P_{i}\right)\;(i=k,\ldots ,m)$. A common used formula for ${\hat{\pi }}_{0}$ is

(5) $$\begin{array}{c} {\hat{\pi }}_{0}=\frac{\left(\text{Number}\;\text{of}\;P\;\text{values}>\lambda \right)}{\left(m\left(1-\lambda \right)\right)} \end{array}$$

for a certain choice of *λ* [11]. Simple choices of *λ* such as 0.5 or 0.75 are often used. Note that this standard estimation procedure does not consider the heterogeneity in population.

## 4. A Bias of the Standard FDR Estimate

Latent variables have been introduced for various purposes in multiple testing framework. Friguet et al. [12] and Leek and Storey [13] considered them as a source of dependence among genes. In this paper we introduce latent variables as a source of heterogeneity and design a latent group model leading to a depleted *P* value distribution near 0. With practical applications in mind, we will adopt terminologies from two-sample microarray studies for cancer. Our toy model is already graphically represented in Section 2. There are two unobserved groups (molecular subtypes 1 (MS1) and 2 (MS2)) and two observed groups (relapse yes (*RY*) and relapse no (*RN*)). Genes affecting relapse within a MS are marked black and genes identifying MS are marked dark gray. More details to generate Figure 1 are as follows.

(i) For most genes, we choose one MS randomly with probability 0.5 and generate background effects from *N*(*μ*/2,1). For other MS, we generate background effects from *N*(0,1). These genes are used to define specific molecular subtypes (MS1 and MS2) and are undiscriminating for the two observed groups (*RY* and* RN*).
(ii) Some genes affect relapse within a MS. After choosing one MS with probability 0.5, we generate background effects from *N*(*μ*/2,1). Then, we add signal effects generated from *N*(*μ*_0_/2,1) for* RY* and *N*(−*μ*_0_/2,1) for* RN*, respectively. For other MS, we generate background effects from *N*(0,1).

**Figure 2:**
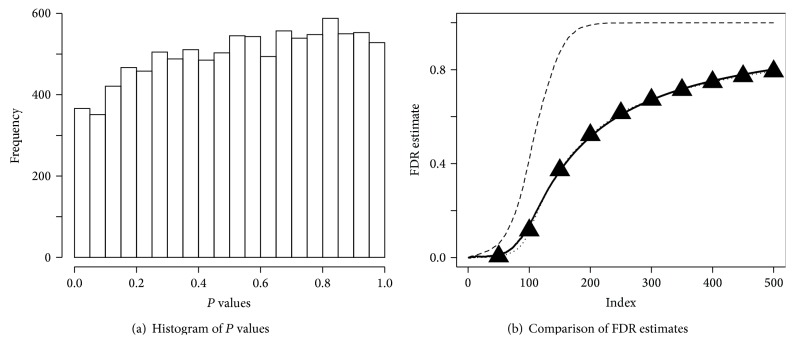
(a) *P*  value = *P*(|*T*| ≥ |*t*_obs_|), where *T* is a generic two-sample *t*-statistic and *t*_obs_ is an observed *t*-statistic and (b) average from 50 simulations: true false discovery proportion (FDP) (solid), standard estimate (dashed), and proposed procedure (dotted). The dotted line coincides with the solid, so it is additionally marked with triangles.

Consider genes defining MS and highly expressed in MS1. Then, we have the following ANOVA representation:

(6) $$\begin{array}{c} Y_{ij}=\left(\frac{\mu }{2}\right)I_{\left(j\in \text{MS}1\right)}+\varepsilon_{ij}, \end{array}$$

where *i* is the index for gene, *j* is the index for patient, and *ε*_*ij*_ ~ *N*(0,1). For relapse-related genes within MS1, we have the following ANOVA representation:

(7) $$\begin{array}{c} Y_{ij}=\left(\frac{\mu }{2}\right)I_{\left(j\in \text{MS}1\right)}+\left(\frac{\mu_{0}}{2}\right)I_{\left(j\in RY\right)}+\left(-\frac{\mu_{0}}{2}\right)I_{\left(j\in RN\right)}+\varepsilon_{ij}^{*}, \end{array}$$

where *ε*_*ij*_^∗^ ~ *N*(0,2). In contrast to our model, Efron [7] considered the following model:

(8) $$\begin{array}{c} Y_{ij}=\left(\frac{\mu_{0i}}{2}\right)I_{\left(j\in RY\right)}-\left(\frac{\mu_{0i}}{2}\right)I_{\left(j\in RN\right)}+\varepsilon_{ij}, \end{array}$$

where *μ*_0*i*_ ~ *N*(0, *σ* ^2^). Note that this model does not consider unobserved group, and as [7] pointed out, this model can lead to only a dilated null distribution that explains inflation of small *P* values (i.e., false positives). In Figure 2(a), however, our latent group model shows the depletion of small *P* values. Note that (6) dominates the overall shape of the *P* value distribution because it has high proportion in the model.

We now see how the unobserved group in the population induces a bias of standard FDR estimate. As a first step, we compute two-sample *t*-statistic to compare* RY* and* RN*. In* RY*, there are *n*_*y*_ patients, where the half are from MS1 and the other from MS2. In* RN*, there are *n*_*n*_ patients and it has the same structure. Thus,* RY* and* RN* groups consist of two normal distributions having different means. Consider the genes following (6). Let ${\bar{RY}}_{i}=\sum_{j}Y_{ij}1_{\left(j\in RY\right)}/n_{y}$ and ${\bar{RN}}_{i}=\sum_{j}Y_{ij}1_{\left(j\in RN\right)}/n_{n}$. The *t*-statistic to test the null hypothesis (non-DE) is

(9) $$\begin{array}{c} z_{i}=\frac{\left({\bar{RY}}_{i}-{\bar{RN}}_{i}\right)}{\left({\hat{\sigma }}_{i}\sqrt{1/n_{y}+1/n_{n}}\right)}, \end{array}$$

where

(10) $$\begin{array}{c} {\hat{\sigma }}_{i}=\sqrt{\frac{\left(\sum_{j}{\left(Y_{ij}-{\bar{RY}}_{i}\right)}^{2}1_{\left(j\in RY\right)}+\sum_{i}{\left(Y_{ij}-{\bar{RN}}_{i}\right)}^{2}1_{\left(j\in RN\right)}\right)}{\left(n_{y}+n_{n}-2\right)}}. \end{array}$$

Note that, for large *n*_*y*_ and *n*_*n*_, we have

(11) $$\begin{array}{c} {\hat{\sigma }}_{i}{⟶}_{p}\sqrt{1+\frac{\mu^{2}}{16}}, \end{array}$$

because

(12) ∑jYij−RY¯i21j∈RYny =∑jYij−μ/421j∈RYny+op(1) =∑j∈RY⋂MS1Yij−μ/42ny  +∑j∈RY⋂MS2Yij−μ/42ny+op(1) ⟶p0.51+μ216+0.51+μ216 =1+μ216,∑jYij−RN¯i21j∈RNnn =∑jYij−μ/421j∈RNnn+op(1) ⟶p1+μ216.

Meanwhile, the numerator in (9) is

(13) n(RY¯i−RN¯i) =n∑jYij1j∈RYny−∑jYij1j∈RNnn =n∑j∈RY⋂MS1Yij−μ/2ny+∑j∈RY⋂MS2Yijny     −∑j∈RN⋂MS1Yij−μ/2nn−∑j∈RN⋂MS2Yijnn ⟶dN(0,4).

Thus, we have for large *n*

(14) $$\begin{array}{c} z_{i}{⟶}_{d}N\left(0,\frac{1}{\left(1+\mu^{2}/16\right)}\right). \end{array}$$

Since 1 + *μ* ^2^/16 > 1 for any *μ* ≠ 0, the use of standard Gaussian distribution for *z*_*i*_ leads to inflated *P* values. Thus, in (4), ${\hat{\pi }}_{0}$ is overestimated and *R*(*c*) is smaller than it should be. Subsequently, (4) overestimates FDR. If the strength of background signal *μ* becomes larger, the degree of depletion of small *P* values becomes more severe because (14) will be more concentrated at 0 as *μ* increases. Consequently, the heterogeneity induced by the unobserved group makes the *t*-statistics conservative and leads to upward bias of standard FDR estimate as shown in Figure 2(b). In our simulation, we use 10,000 genes and 60 patients, with 30 belonging to each MS. The proportion of genes defining specific MS is 0.99. Within each MS, the number of* RY* and* RN* is assumed to be same for simplicity and we use *μ* = 2 and *μ*_0_ = 3.

## 5. Proposed FDR Estimation Procedure

While performing CAMS, we want to assess whether clustering results are informative or not with respect to a measure based on FDR. Thus, in computing FDR estimate, the population heterogeneity should be addressed properly. Furthermore, when many datasets are considered simultaneously, it is desirable to have a fast and stable algorithm to compute FDR estimate. Reflecting these aspects, we propose a new FDR estimation procedure.

Our starting point is Pawitan et al.'s FDR estimation procedure [9] because it is computationally flexible to accommodate new changes. A similar permutation-based approach to deal with the dependence in computing FDR estimates was developed by [14]. Pawitan et al. [9] explored the variation pattern of the null distribution of test statistics using the singular value decomposition (SVD) when there are correlations between genes. To check the validity of the SVD analysis in our problem, it is needed to confirm whether the main variation pattern of permutation distribution can represent that of sampling distribution.

### 5.1. The Validity of SVD Analysis

We firstly demonstrate the variation pattern of the sampling distributions from the latent group model through the SVD analysis. We partition the range of the observed statistics into *B* equispaced bins with width Δ. Let the histogram-count vector **y** = (*y*_1_,…, *y*_*B*_) be the number of statistics that fall into each bin. Each simulation contributes a single count vector **y**_**i**_. Let **η** be the expected histogram-count vector from standard Gaussian distribution and the *B* × *K* matrix *Y* the matrix of centered count vectors **y**_**i**_ − **η**. *K* is the number of simulations and 50 is used in our example.

**Figure 3:**
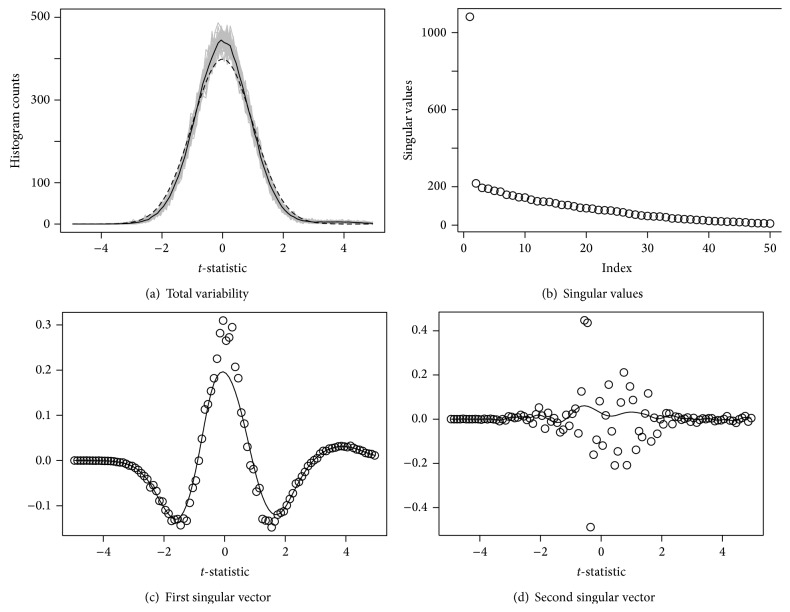
(a) Each simulation contributes a single gray line. The solid black line is the average of 50 simulations, and dashed line is the expected histogram-count vector from *N*(0,1). (b) shows singular values from the singular value decomposition (SVD) of *Y*, and the dots in (c) and (d) are the components of the singular vectors generated by the SVD, and the solid lines are robust smoothing curves.

Figure 3(a) shows the total variability of sampling distributions; the solid line is $\bar{y}$ and the dashed line is **η**. The solid line has higher peak and smaller width than the dashed line, so this is consistent with our analytical findings. To see the variability of **y**_**i**_ − **η**, we perform the SVD of *Y*. The variation is dominated by one large singular value, associated with the pattern seen in the plot of the first singular vector. A consequence of this pattern is that the sampling distribution tends to have a leptokurtic shape compared to the standard Gaussian distribution. Subsequent singular vectors do not have large contributions to the variation.

**Figure 4:**
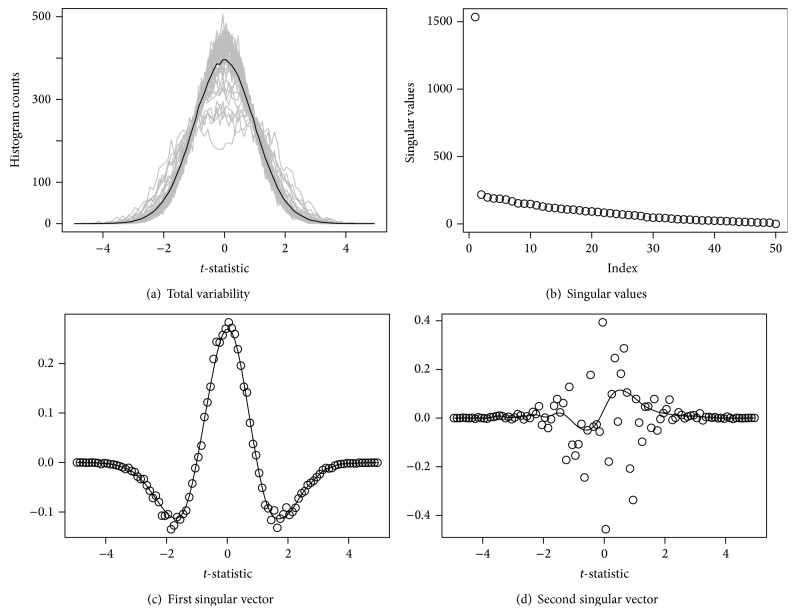
(a) Each permutation contributes a single gray line. The solid black line is the average of 100 permutations. (b) shows singular values from the singular value decomposition (SVD) of *Y* ^∗^, and the dots in (c) and (d) are the components of the singular vectors generated by the SVD, and the solid lines are robust smoothing curves.

In practice, we cannot create real data as in simulation. To circumvent this problem, we use permutation to generate the null distribution, but we first check the variability pattern of the distributions from permutation. Let *X* be a microarray data matrix, let **g** = (*g*_1_,…, *g*_*n*_) be the vector of group labels, and let *g* ^∗^ be a random rearrangement of *g*. With each permuted dataset (*X*, *g* ^∗^), we compute test statistics. So each permutation contributes a single count vector **y**_**i**_^∗^. Let ${\bar{y}}^{*}$ be the mean vector of **y**_**i**_^∗^ over *K* permutations and the *B* × *K* matrix *Y* ^∗^ the mean-corrected matrix of count vector **y**_**i**_^∗^. The SVD results of *Y* ^∗^ are reported in Figure 4.

Figure 4(a) shows the total variability of the distribution over permutations, and the solid line is the average of the permuted null distributions. In Figure 4(b), the first singular value is dominating others and Figure 4(c) shows that the pattern of the first singular vector from *Y* ^∗^ is very close to that from *Y*. This implies that the main variation of permuted distributions explains that of the sampling distributions well, so the SVD analysis for permuted data is valid under our latent group model.

Since we have the validity of the SVD analysis, Pawitan et al.'s method [9] can be adopted to correct the overestimation by unobserved group. We assume that the observed statistics *z* follow a mixture model (1). They suggested to fit

(15) $$\begin{array}{c} y\sim \text{Poisson}\left(m\Delta f\left(z\right)\right), \end{array}$$

where

(16) $$\begin{array}{c} f\left(z\right)=\pi_{0}\left(\phi_{0}\left(z\right)+b\phi_{1}\left(z\right)\right)+\left(1-\pi_{0}\right)f_{1}\left(z\right), \end{array}$$

where *f*_0_(*z*) = *ϕ*_0_(*z*) + *bϕ*_1_(*z*), *ϕ*_0_(*z*) is the average of null distributions over permutations, and *ϕ*_1_(*z*) is the first singular vector of *Y* ^∗^. In this paper, the parameter *b* captures the variation of the null distribution due to the heterogeneity by unobserved group. The original computing procedure is given as follows.

(1) Perform *K* permutations of group labels. Each permuted dataset generates a histogram-count vector **y** ^∗^.
(2) Compute the predictor *ϕ*_0_ from the average vector ${\bar{y}}^{*}$ by scaling so that it integrates to 1.
(3) Construct a matrix *Y* ^∗^ from the **y** ^∗^s. Compute the predictor *ϕ*_1_ from the smoothed first singular vector *u*_1_.
(4) Since *f*_1_ is unknown, the regression is performed in two steps. First, fit the reduced model **y**~ Poisson(*μ* = *m*Δ*f*), where
  (17) $$\begin{array}{c} f=\beta_{0}\phi_{0}+\beta_{1}\phi_{1}, \end{array}$$
 and compute the residual vector $r=y-\hat{y}$. Estimate *f*_1_ by smoothing the residual vector **r**/*m*Δ as a function of *z*.
(5) Fit the full model (16)
  (18) $$\begin{array}{c} f=\beta_{0}\phi_{0}+\beta_{1}\phi_{1}+\beta_{2}f_{1}, \end{array}$$
 and reestimate the full set of coefficients (*β*_0_, *β*_1_, *β*_2_).

The coefficient of *ϕ*_0_ becomes the estimate for *π*_0_. Given estimates of parameters, *P* values inflated by the heterogeneity are corrected by using the following definition:

(19) $$\begin{array}{c} P\;\;\text{value}=\int_{\left|z\right|\geq \left|z_{\text{obs}}\right|}\hat{f_{0}}\left(z\right)dz, \end{array}$$

where $\hat{f_{0}}(z)$ is the null density estimate corrected by the first singular vector. For the FDR estimate, we have

(20) $$\begin{array}{c} \hat{\text{FDR}}\left(c\right)=\frac{m{\hat{\pi }}_{0}\int_{|z|>c}{\hat{f}}_{0}\left(z\right)dz}{\sum_{i}I\left(\left|z_{i}\right|>c\right)}. \end{array}$$

(Strictly speaking, this is an FDP estimate rather than an FDR estimate.) Simulation studies show that this estimate has a negligible bias (Figure 2(b)). It may be possible to improve the null density estimate further using the second singular vector in some cases, but we will not attempt this here.

### 5.2. Improved Algorithm for Many Datasets

CAMS generates many subtypes. Since not all the subtypes are meaningful, it is needed to assess each of them quickly. In particular, for the subtypes showing the depletion of small *P* values, it is desirable to apply our FDR procedure to address such depletions.

One measure to assess subtypes from CAMS is the number of genes having FDR < *c*, where *c* is a suitably chosen small value; we use *c* = 0.1 in our examples and call this measure *N*01.  Figure 1(c) shows that *N*01 is obtained by counting the number of genes with FDR < 0.1. To compute *N*01 for many datasets, the previous procedure becomes

1. computationally intensive: the permutation step takes long time;
2. unstable: some null density estimates have negative values.

To increase computational speed, we note that $\hat{f_{0}}(z)$ from the SVD analysis is empirically well approximated by $N(0,{\left(1-b/\sqrt{2}\right)}^{2})$ for |*b* | <0.2, which can be checked before the permutation step. But this approximation does not seem to be reliable when |*b* | >0.2. Furthermore, the null density estimates often have negative values when *b* is large and this leads to a numerical problem in estimating FDR. Thus, we propose a more stable algorithm to find good approximation to the null density. The main idea is to pick up a few vectors **y**_*i*_^∗^ that are closest to the histogram counts of the observed test statistics **y** with respect to a certain metric. To emphasize goodness of fit at the center of the distribution, we use

(21) $$\begin{array}{c} \text{Dist}\left(y,y_{i}^{*}\right)={\left(y-y_{i}^{*}\right)}^{T}W_{y}\left(y-y_{i}^{*}\right), \end{array}$$

where *W*_**y**_ = Diag(**y**) as a distance measure. This distance measure gives larger weights to the central part of the histogram. We find top 5 curves that minimize (21) and use their average as $\hat{f_{0}}(z)$. For simplicity we estimate *π*_0_ with (5). The resulting procedure thus becomes as follows.

(i) Before the permutation step, approximate $\hat{f_{0}}(z)$ by $N(0,{\left(1-b/\sqrt{2}\right)}^{2})$ and obtain *ϕ*_0_ and *ϕ*_1_ using known functional forms [15]. After fitting (16) as described in Steps 4 and 5 in the previous section, check whether |*b* | <0.2 or not. If |*b* | <0.2, compute FDR estimate.
(ii) In the case of |*b* | >0.2, perform *K* permutations and compute (21) for each permuted dataset. Find the top 5 curves that minimize (21) and take the average as the null density estimate. Estimate *π*_0_ using formula (5).

## 6. Assessing the Clustering Results from CAMS

CAMS can generate a practically unlimited number of candidate subtypes by permuting the gene probes for doing extensive search. If a subtype is depleted in small *P* values, it is desirable to assess it with *N*01. To see the proportion of subtypes requiring such assessment, we define the ratio of low *P* value areas as

(22) $$\begin{array}{c} \text{Ratio}\left(\lambda \right)=\frac{\sum_{i}^{m}1_{\left(P_{i}\leq \lambda \right)}}{m\lambda }, \end{array}$$

where *λ* = 0.2 is used in practice. The denominator corresponds to the expected number of *P* values less than *λ* when the null hypothesis holds. We regard Ratio < 1 as indicating the targeted situation (the depletion of small *P* values). When the whole set of patients shows the depletion, we often observe high proportion of potential subtypes with Ratio < 1, so it is safe to use *N*01 as a default assessment measure.

We provide an implementation of the proposed method as an *R* package at http://fafner.meb.ki.se/personal/yudpaw/. Two necessary inputs for the implementation are gene expression data matrix and corresponding group vector (a clinical outcome such as disease outcome, e.g., relapse indicator). To enable further analysis when there is auxiliary information such as survival time, the software stores the following results:

(i) genes defining a cancer subtype,
(ii) patient IDs that belong to a subtype,
(iii) *N*01 and respective *P* value.

Note that we may have high *N*01 by chance because several optimization procedures (e.g., the biclustering procedure) are performed before computing *N*01. To address this point, we randomly permute group labels of each subtype *N*_*p*_ times and compute *N*01 based on the permuted data (*N*01_perm_).

Then, we compute a standardized statistic of *N*01 for *i*th subtype:

(23) $$\begin{array}{c} z_{i}=\frac{N01_{i}-\bar{N01_{i}}}{s_{i}}, \end{array}$$

where $\bar{\text{N}01_{i}}$ and *s*_*i*_ are the mean and standard deviation of *N*01_*i*_ and *N*01_perm_'s. *N*_*p*_ = 50 is used in practice. Likewise, we standardize *N*01_perm_. This standardization enables us to have precise estimate for *P* value and reasonable resolution for estimating FDR. After stacking all the standardized statistics in a vector *z*_perm_, the *P* value of *N*01 for *i*th subtype is defined as

(24) $$\begin{array}{c} P\text{-valu}{\text{e}}_{i}=\frac{\sum_{k}I\left(z_{i}\leq z_{\text{perm},k}\right)}{K}, \end{array}$$

where *K* is the length of *z*_perm_ and *z*_perm,*k*_ is the *k*th element in *z*_perm_. Thus, the subtype with large *P* value (24) will not be considered as an interesting cancer subtype even though it has high *N*01.

To find clinical implication of the subtype, we evaluate the prognostic signature in the subgroup of patients using the logistic regression with L1 penalty. We first classify patients belonging to the subgroup into good and poor prognosis groups based on cross validated probabilities of being relapsed patients from the logistic regression. Then, the strength of the prognostic signatures from the logistic regression is assessed by computing the survival difference between good and poor prognosis groups and the area under the operating characteristic curve (AUC).

## 7. Real Data Analysis

### 7.1. Chemores Data Example

Lung cancer is one of the most prevalent and deadliest cancers. Human lung cancers are classified into two major subtypes, small cell lung cancer (SCLC) and non-small cell lung cancer (NSCLC). NSCLC, which accounts for around 80% of all primary lung cancers, is a known heterogeneous group and its prognosis is generally poor [16]. In the current clinical practice, it is difficult to perform histopathological classification with small biopsies [17]. In order to improve the selection of patients who most likely will benefit from adjuvant chemotherapy (ACT), there is an urgent need to establish new diagnostic tools.

**Figure 5:**
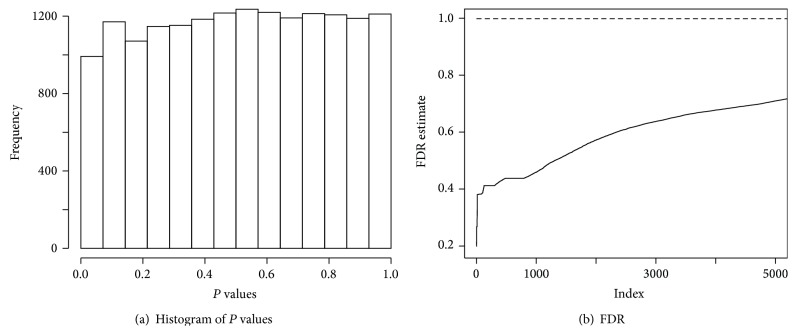
(a) *P* value distribution of two-sample *t*-statistics for detecting differentially expressed genes from a lung cancer data comparing relapse versus no relapse and (b) the corresponding false discovery rate (FDR) estimate. In (b), the dashed line is the standard FDR estimate and the solid line is from our proposed procedure. The *x*-axis in (b) denotes the ranking of genes where higher ranking corresponds to higher statistical significance.

In this view, a study was organized by the Chemores initiative, which became an EU funded (FP6) Integrated Project involving 19 academic centers, organizations for cancer research, and research-oriented biotechnology companies in 8 European countries. Tissue samples from a cohort of 123 patients who underwent complete surgical resection between 30 January 2002 and 26 June 2006 are analyzed. All the patients belong to NSCLC and 59 patients experienced a relapse. This group of patients represents a heterogeneous group of lung cancers. We assayed the samples for gene expression, performed using dual-color human array from Agilent containing 41000 gene probes; a dye-swap of tumor versus normal lung tissue from same individual was employed for each sample and the log-ratio values were combined by averaging (the dataset is available at http://www.ebi.ac.uk/arrayexpress/experiments/E-MTAB-1132).  Figure 5(a) shows the depletion in small *P* values of the two-sample *t*-statistics for the 41000 gene probes.  Figure 5(b) shows the corresponding pessimistic standard FDR estimate by [11] (dashed line). Thus, to take into account the heterogeneity issue properly, CAMS is needed here. Two inputs for implementing CAMS are a gene expression matrix and a relapse indicator.  Table 1 shows a summary of output. The first column of this output contains unique names of subtypes. The second and third columns tell how many genes are involved in defining each subtype and the number of patients in the subtype. The *P* values in the last column are computed using (24). The full lists of genes and patients can be identified by SubtypeID.

**Figure 6:**
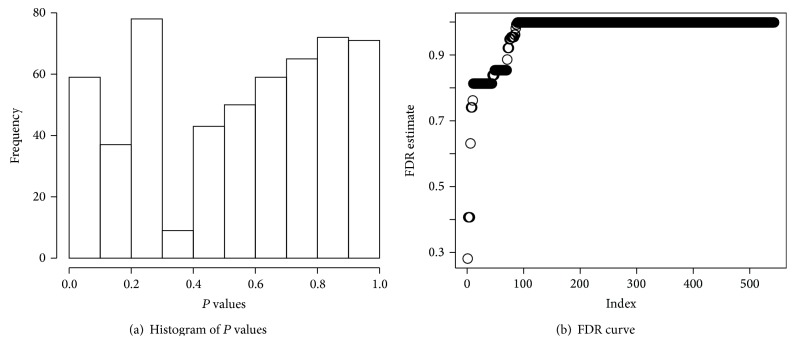
(a) Histogram of *P* values for *N*01 from a lung cancer data and (b) the corresponding false discovery rate (FDR) estimate.

**Figure 7:**
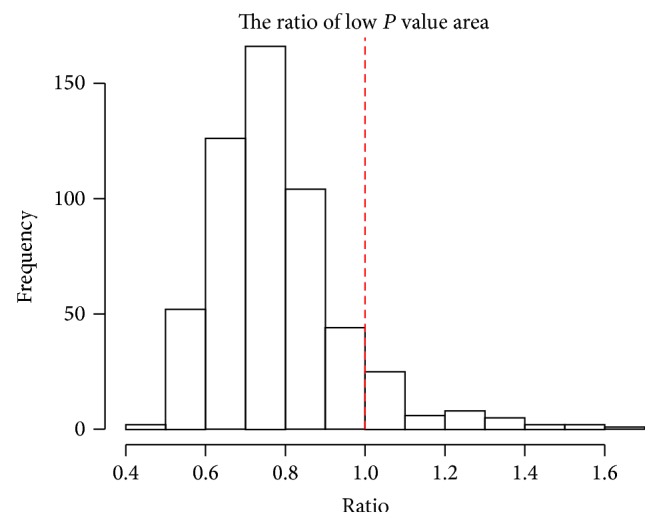
The proportion of subtypes showing depleted *P* value distribution from our clustering results is 0.908 (left side of vertical dashed line).

To reduce the computation time further, we consider filtering out uninteresting cases in the first stage. We compute *N*01 through the FDR based on the normal approximation only. We call this *N*01_0_. If *N*01_0_ is small, we skip the remaining procedure and go to search for next subtype.  Figure 6(a) shows histogram of *P* values for *N*01 after filtering out the uninteresting cases having *N*01_0_ ≤ 2. The standard FDR estimate is given in Figure 6(b), showing some interesting subtypes. In this analysis, we compute the proportion of subtypes showing depleted distributions with (22) and it is 0.908 (Figure 7). Therefore, *N*01 is essential in assessing the quality of each subtype.

**Figure 8:**
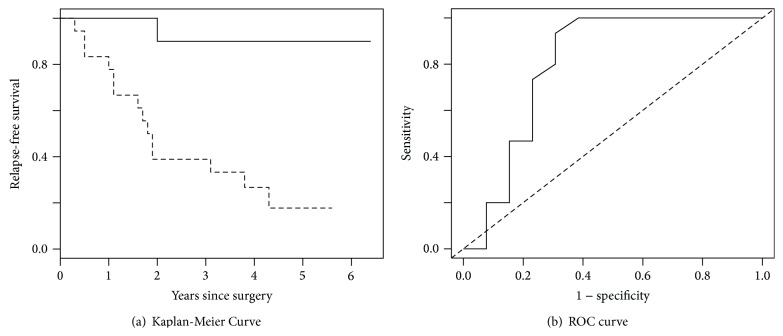
(a) Kaplan-Meier curves of good and poor prognosis groups for a promising subtype and (b) receiver operating characteristic (ROC) curve.

From the top list of subtypes, one promising subtype is further analyzed using survival information to compute the appropriate prognostic signature for that subtype. To deal with large number of predictors (genes) we use logistic regression with L1 penalty [18] where the relapse status is the response variable. The cross validated probability of being a relapsed patient is computed from the leave-one-out cross validation, and the poor prognosis group is defined as the patients having the probability ≥0.5. To assess the strength of the prognostic signatures from the logistic regression, we compute the survival difference between good and poor prognosis groups. In Figure 8(a), the Kaplan-Meier curves of relapse-free survival show big difference between those two groups.  Figure 8(b) shows operating characteristic curves for identifying relapse during follow-up. The area under the curve (AUC), computed under leave-one-out cross validation, is 0.806.

### 7.2. Bild et al.'s Data Example

**Figure 9:**
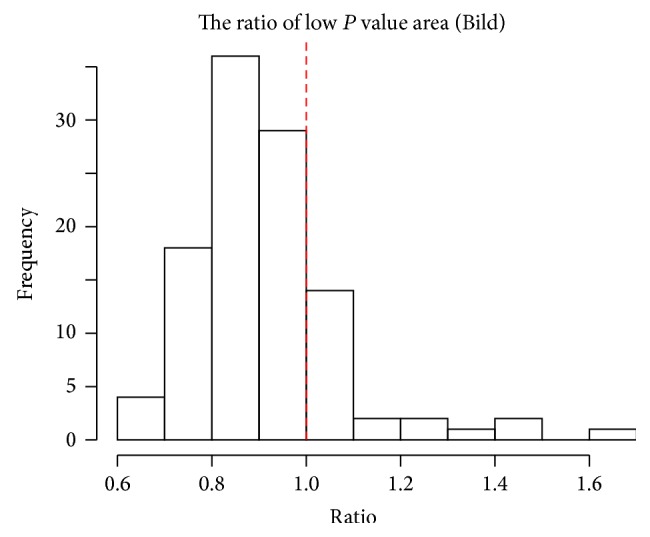
The proportion of subtypes showing depleted *P* value distribution from our clustering results is 0.798 (left side of vertical dashed line).

As another application, we use lung cancer data by Bild et al. [19]. Their research purpose was to identify gene expression signatures of human cancers that reflect the activity of a given pathway. The gene expression dataset for lung cancer consists of 53 squamous cell carcinomas (SCC) and 58 adenocarcinomas (AC), so we expect that the group of patients represents a heterogeneous group. Among 58 relapsed patients, 26 and 32 patients belong to SCC and AC, respectively. The expression dataset was obtained using Human U133 2.0 plus arrays (Affymetrix) containing 56475 gene probes. It is available at http://www.ncbi.nlm.nih.gov/geo/query/acc.cgi?acc=GSE3141. For the downstream analysis, we normalized the dataset for each patient to have zero mean after taking logarithm. The same procedures as described in analyzing Chemores data were applied to the normalized data. The proportion of subtypes showing depleted distributions is 0.798, so *N*01 is crucial in assessing the quality of each subtype. See Figure 9. Likewise in the previous section, further survival analysis can be done, but we omit the results here for brevity.

## 8. Discussion and Conclusions

In this paper, we proposed an extensive clustering algorithm to find cancer subtypes and have addressed the heterogeneity issue induced by the unobserved group to assess the resulting subtypes appropriately. The unobserved group creates a serious conservative bias problem when standard FDR estimation is used, but our proposed FDR estimation method resolves it. SVD is used as a tool for discovering the effect of heterogeneity on the null distribution of the test statistics. In particular, when many datasets are considered simultaneously, we develop a much faster and more stable FDR estimation algorithm than the method in [9].

Although we focus only on the heterogeneity issue in this paper, Efron's three issues [7] should be considered simultaneously in high-throughput data analysis. It is difficult, however, to distinguish genes with small effects from correlation effects because both can produce similarly wide distributions of the test statistic. We also expect that there is some confounding between the heterogeneity effect and the above two effects. Thus, careful joint approaches for dealing with the three issues are required. For example, Pawitan et al. [8] showed that it is possible to get less bias by estimating *π*_0_ and *f*_1_(*z*) using a joint estimation method. This issue needs further investigation.

Recently, several biclustering algorithms have been proposed for gene expression data, and a comparative study was performed in [20]. They pointed out that performance on synthetic datasets did not always correlate with that on real datasets and no algorithm is uniformly the best under different environments. Considering this point, CAMS is also expected to have its own weakness and strength. Thus, it is needed to study when CAMS performs well compared to other biclustering methods. On the one hand, it is possible to embed existing biclustering algorithms into CAMS with some modification. Then, we can compare performances of various biclustering methods when subtypes are assessed by *N*01.

In addition to the above issues, there are still many scientific questions to be considered here. For example, should two similarly constructed clusters be combined or remained separate? How can we assign an independent test sample to newly constructed subtypes? A practical method for dealing with these scientific problems will require further research.

## Figures and Tables

**Algorithm 1:**
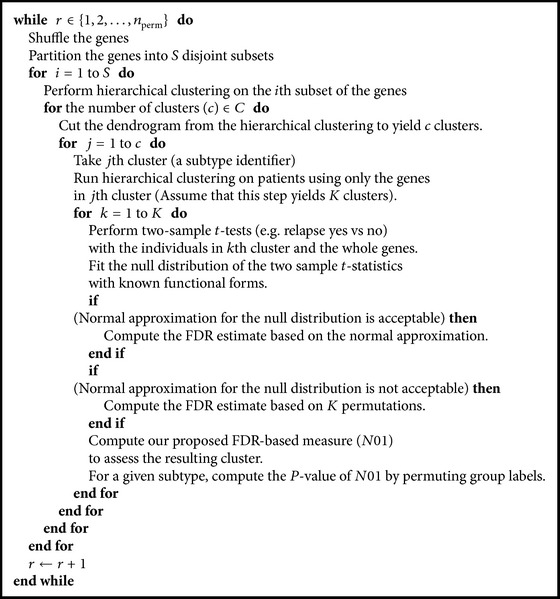
CAMS.

**Table 1:** The output from *R* package.

Subtype_ID	Genes_in_clusters	Patients_in_subtype	*N*01	*P* value^∗^
⋮	⋮	⋮	⋮	⋮
6	1535	69	17	0.020
7	124	28	23	0.020
⋮	⋮	⋮	⋮	⋮

^∗^The *P* value of *N*01 for *i*th subtype is computed by using (24).
